# Editorial: Optimizing outcomes and addressing adversities of immunotherapy in lung cancer

**DOI:** 10.3389/fonc.2023.1277542

**Published:** 2023-09-08

**Authors:** Taiseer Al-Rajabi, Jarushka Naidoo, Jun Zhang

**Affiliations:** ^1^ University of Kansas, Lawrence, KS, United States; ^2^ Sidney Kimmel Comprehensive Cancer Center, Johns Hopkins Medicine, Baltimore, MD, United States; ^3^ Division of Medical Oncology, Department of Internal Medicine, University of Kansas Medical Center, Kansas City, KS, United States; ^4^ Department of Cancer Biology, University of Kansas Medical Center, Kansas City, KS, United States

**Keywords:** immune checkpoint inhibitors, immunotherapy, non-small cell lung cancer (NSCLC), immune related adverse effects (irAEs), cancer immunotherapy

Lung cancer is the leading cause of cancer-related death worldwide. Lung cancer is categorized into several histologic subtypes, principally small cell lung cancer (SCLC) and non-small cell lung cancer (NSCLC), of which NSCLC accounts for 85% of cases. NSCLC is mainly comprised of squamous cell carcinoma, adenocarcinoma, and large cell carcinoma (Yang et al.) Because of the poor survival associated with NSCLC, it is imperative to identify efficacious new treatments with the goal of improving outcomes as well as minimizing side effects for all affected patients. Among recent treatments, immune checkpoint inhibitors have been a major class of therapy that has changed how lung cancer is treated- by bolstering the immune response.

This Research Topic in Frontiers in Oncology, “*Optimizing Outcomes and Addressing Adversities of Immunotherapy in Lung Cancer*,” is aimed at providing insight into clinical decision making as it applies to the use of immunotherapy for lung cancer. A total of 16 publications are included in this Research Topic. Herein, we aim to summarize these studies and discuss how variables in biology, tumor response, progression, and side effects can potentially influence treatment decisions.

Immune checkpoint inhibitors (ICIs) in the treatment of NSCLC are used to enhance T cell response against cancer cells in the immune system. Programmed cell death protein 1 (PD-1) is a receptor, which is expressed on the surface of activated T cells. If PD-1 binds to its ligand (PD-L1), the cell possessing the ligand may escape its destruction, even if it is cancerous. There are multiple ways to utilize ICIs in the treatment of NSCLC; they can be used as a monotherapy or in combination with another therapy. Each treatment discussed will highlight the benefits of ICIs in patients of various medical conditions and lifestyles. Factors such as age, ethnicity, tumor mutational burden, and comorbidities are possible examples of what can affect the prognosis. Two studies (Huang et al.; Shiotsu et al.) explored the effect of pembrolizumab on NSCLC. Pembrolizumab is an Immune checkpoint inhibitor drug that serves as a humanized IgG4 monoclonal antibody for the PD-1 protein. When evaluated on a patient population who had poor performance status (PS) or were elderly, pembrolizumab monotherapy was found to be an effective 1^st^ line treatment for those with PD-L1-positive advanced NSCLC (Shiotsu et al.). Huang et al compared pembrolizumab to the angiogenesis inhibitor bevacizumab. Bevacizumab weakens angiogenic behaviors of cancer by promoting the normalization of tumor vessels and reducing the formation of new blood vessels. The results showed that both pembrolizumab and bevacizumab are effective treatment options, especially when combined with another systemic therapy such as chemotherapy. However, in PD-1-positive patients, the results showed that immunotherapy was clearly superior.

ICIs are appealing in that the effect comes with less toxicity when compared to conventional systemic treatments such as chemotherapy. Using meta-analysis, Yang et al’s comparison study showed that in the second line setting for advanced/metastatic NSCLC, ICIs were superior to the chemotherapy drug, docetaxel. Docetaxel has less efficacy and more toxicities. ICIs were found to have a better OS and PFS of NSCLC patients when compared to docetaxel (Yang et al.).

Though effective as a monotherapy, ICIs can be more beneficial when used in conjunction with other treatments such as chemotherapy. Two studies investigated the potential of ICIs as a neoadjuvant treatment. Shi et al. confirmed the usefulness of PD-1 inhibitors in the treatment of resectable squamous NSCLC with chemotherapy. Although exploring a relatively small population size (n=63), the majority of the patients in this study (66.7%) demonstrated a major pathologic response (MPR), including 39.7% resulted in pathologic complete response (pCR), with low risk of toxicity when treated with PD-1 inhibitors and chemotherapy. Using another humanized monoclonal PD-1 antibody, camrelizumab, Li et al showcased the potential of camrelizumab in the neoadjuvant setting for resectable IIIA squamous NSCLC, especially in combination with chemotherapy. These studies confirmed the value of using ICIs in the neoadjuvant setting for resectable NSCLC (1).

Though using ICI drugs over other treatments presents the benefit of low toxicity, the emergence of immune-related adverse events (irAEs) can occasionally become life threatening to patients. Because of this, predictive markers for irAEs are greatly needed when ICIs are used. For example, a study by Koh et al. was conducted to evaluate the relation between proteins YTHDF1 and YTHDF2, and ICIs. YTHDF1 and YTHDF2 were found to negatively affect the expression of CD8 and CD4 in T cells, and that groups with low expression of both proteins responded better to PD-1/L1 inhibition. Another study by Lan et al discovered the use of CURB65 scores to predict the incidence of irAEs, primarily the checkpoint inhibitor-associated pneumonitis (CIP) in patients receiving immunotherapy. Among 28 enrolled patients with CIP, they found mortality after onset of CIP was consistently higher in the high-CURB65 group than in the low-CURB65 group, and higher CURB65 score positively correlated with higher grade of CIP. CURB65 therefore could be further evaluated as a potential predictive biomarker for CIP. Another relevant signal for irAEs has been found in cytokines, which are molecules that interact with the immune system. Cytokines’ presence in the bloodstream and tendency to appear during response makes them a candidate for potential biomarkers of irAEs or treatment response. A study by Zhao et al searched for positive correlations between a defined cytokine panel (IL-1β, IL-2, IL-4, IL-5, IL-6, IL-8, IL-10, IL-12, IL-17, IFN-α, IFN-γ, TNF-α) and irAEs. A positive association with occurring irAEs was found with cytokines IL-1β and IL-2 levels in peripheral blood. The levels of IL-5, IFN-α and IFN-γ during ICI treatment were also correlated with irAEs. In analyzing clinical response, only levels of IL-6, IL-8, IL-10, and IL-17 levels during treatment were positively associated. A separate case study by Yin et al. extended on the investigation of the role of IL-6 during the incidence of myocarditis. During the patient’s treatment, IL-6 rose to thousands of times its normal level while multiple irAEs were present. The level of this cytokine only decreased when steroids were administered to counter the irAEs. These results show that cytokine molecules are immune-related, and a precise understanding of their dynamic composition might be used in predicting treatment response and/or irAEs.

To further characterize biological factors that could impact immunotherapy response, a study by Nakagawa and Kawakami was developed to analyze previous reports on ICI treatment in varying patient populations. They concluded that patients with driver mutations on the EFGR or ALK genes have poorer reactions to ICI therapy, thought to be caused by a lowered tumor mutational burden. Conversely, patients with mutations on the KRAS or BRAF gene received greater benefit from ICI therapy. Finally, co-mutation SKT11/LKB1 with the KRAS mutation has been shown to correlate with lower PD-L1 expression. All in all, driver mutations may have varying effects on treatment depending on the affected gene(s). There are also situations that emerge to affect the treatment of NSCLC, such as metastases in advanced cases. Liver metastases are generally associated with poorer outcomes and have no established optimal treatment. Conversely, brain metastases have a clear treatment decision, and should be treated as soon as possible with radiation. Another emergent effect is pleural effusion, which is associated with worsened outcomes. An article published by Chen et al collected data to correlate the time between neoadjuvant immunotherapy and surgery, known as time-to-surgery (TTS) with treatment outcomes in the early-surgery group, the standard-surgery group, and the delayed-surgery group. They concluded that TTS has no relevant influence on the feasibility and safety of surgery in neoadjuvant immunochemotherapy. It is recommended to combine bevacizumab and ICI therapy to treat pleural effusion, but there is scarce literature published on this topic. A patient’s elderly status does not have much correlation with treatment outcomes, but a poorer prognosis often comes with poor PS and comorbidities (Nakagawa & Kawakami). Another study confirmed this, as patients with comorbid burden likely have a weakened physical status from hospitalization. This correlates comorbidities with poorer clinical outcomes (Young et al.). Though often excluded from most studies involving ICIs, patients with interstitial lung disease have worse survival (Nakagawa & Kawakami).

To account for the many variables that may help or hinder the patient’s prognosis, optimization of treatments is necessary to discover safer and less strenuous solutions. Combining ICIs with chemotherapy has shown prolonged survival, but other combination therapies may provide an equally effective result with less toxicity. To extend on this, a study by Martin and Enrico was initiated to investigate other combinations using immunotherapy and discuss the results of multiple therapies. When ICIs were combined with chemotherapy, this combination significantly prolonged the median progression-free survival (PFS) compared with chemotherapy alone. Immunotherapy can also be a main treatment, and in first-line immunotherapy, nivolumab plus ipilimumab significantly improved OS relative to chemotherapy alone. Antiangiogenic agents such as bevacizumab have also been reported to be efficacious when used alongside ICIs. Antiangiogenic agents also synergized with multi-kinase inhibitors such as lenvatinib, cabozantinib, and axitinib. PD-1/L1 blocking agents have been reported to work well with drugs that target LAG3, which is another immune checkpoint expressed with unfavorable clinical outcomes. Martin and Enrico, in their review pointed out that utilizing relatlimab and nivolumab has proven effective in treating metastatic or unresectable melanoma. Other immune checkpoints of T cells exist, such as VISTA and TIM-3, but each have an accompanying drug to be used alongside ICIs for similar results to PD1/L1 blocking. Finally, Oncolytic virus therapy may serve as a novel strategy that uses immunogenic cell death to spur the immune system into a desired response.

A novel area in the field of immunotherapy in which there is no current consensus, is regarding hyperprogressive disease (HPD). Although lacking a precise definition, it was originally described as disease progression at the first evaluation and at least two-fold tumor growth rate increase between pre-immunotherapy and immunotherapy period (2). One study by Britt et al sought to analyze HYD to compile the many speculations on its details. Britt et al described HYD as a rapid acceleration of tumor growth following ICI therapy, where cancer lesions would show an increase of two-fold or higher per RECIST 1.1 criteria, or, 50% or higher increase in tumor burden compared to pretreatment imaging, despite having been treated. The mechanism of such clinical presentation is largely unknown with conflicting accounts (3). To identify a proper biomarker for predicting HYD, the authors concluded that more studies should be devoted to the relation of HYD in T cell regulation, changes in the tumor microenvironment, and genomic changes (Britt et al.).

Finally, two studies in this series explore the pitfalls of immunotherapy across different ethnicities. For example, in comparison to the European and American populations, the Asian population exhibits a unique disease prognosis due to having a differing tumor mutation burden (TMB). There is also a clear difference in the survival between hispanic populations and non-hispanic white populations. Sun et al. defined TMB as a biomarker that can predict the response to ICI therapy, but compared to western populations, it was concluded that the TMB values of Asian populations seem decreased in comparison. Somatic-germline-zygosity is an algorithm to calculate TMB, and by calibrating it to Asian populations, TMB cut-off was found to be seven mut/Mb instead of ten mut/Mb in European and American populations. Having unreliable biomarkers causes a disparity between the two populations. Raez et al reported this disparity of treatment with a different cause. Of the patients with locally advanced stage III NSCLC, non-Hispanic white (NHW) patients had better survival outcomes when compared to Hispanics. As a retrospective study, the explanation could be from multiple differences between Hispanics vs. NHW, including access to optimal second-line therapy or follow-up, which is a crucial part contributing to overall survival.

The combined efforts of these studies map out the ever-expanding effects of immunotherapy on innovating treatment of NSCLC. As new techniques are developed, more information must be gained to each minute detail, or the influence of said treatments cannot be gauged accurately. Novel studies will continue to come out in hopes of discovering combinations with less risk, as well as reasonable counters to the side effects. In the background, algorithms for quantifying biomarkers will also be worked on so members of different populations will have the same access to suitable treatments.

## Author contributions

JZ: Supervision, Writing – review & editing. TA-R: Formal Analysis, Investigation, Writing – original draft, Writing – review & editing. JN: Writing – review & editing.
